# Impaired glucose tolerance in women with *BRCA1* versus *BRCA2 pathogenic or likely pathogenic variants*: Results from a prospective cohort study

**DOI:** 10.1007/s10689-026-00570-3

**Published:** 2026-05-14

**Authors:** Giovanni Grandi, Claudia Piombino, Giovanna Sighinolfi, Elena Barbieri, Marta Venturelli, Chiara Melotti, Rebecca Lippi Bruni, Valentina Grisendi, Riccardo Cuoghi Costantini, Mina Grippa, Emma Zattarin, Elena Tenedini, Elisabetta Razzaboni, Giulia Brigante, Roberto D’Amico, Benedetta Nanni, Renata Menozzi, Laura Cortesi, Massimo Dominici, Antonio La Marca, Angela Toss

**Affiliations:** 1 Obstetrics and Gynecology Unit, Azienda Ospedaliero-Universitaria di Modena, Modena, Italy; 2 Department of Medical and Surgical Sciences, University of Modena and Reggio Emilia, Modena, Italy; 3 Division of Oncology, Department of Oncology and Haematology, Azienda Ospedaliero- Universitaria di Modena, Modena, Italy; 4 Unit of Statistical and Methodological Support to Clinical Research, Azienda Ospedaliero- Universitaria di Modena, Modena, Italy; 5 Unit of Medical Genetics, Azienda Ospedaliero-Universitaria di Modena, Modena, Italy; 6 Department of Laboratory Medicine and Pathology, Diagnostic Hematology and Clinical Genomics Unit, Azienda Ospedaliero-Universitaria di Modena, Modena, Italy; 7 Hospital Psychology Unit, Azienda Ospedaliero-Universitaria di Modena, Modena, Italy; 8 Department of Biomedical, Metabolic and Neural Sciences, University of Modena and Reggio Emilia, Modena, Italy; 9 Unit of Endocrinology, Department of Medical Specialties, Azienda Ospedaliero-Universitaria of Modena, Modena, Italy; 10 Division of Metabolic Diseases and Clinical Nutrition, Department of Specialistic Medicines, Azienda Ospedaliero-Universitaria of Modena, Modena, Italy

## Abstract

**Background:**

Estrogens exert a beneficial effect on metabolism. Women carrying *BRCA* likely pathogenic/pathogenic variants (LP/PV) are at increased risk of premature menopause and may therefore be at higher risk of developing metabolic disorders later in life. In this single-center prospective cohort study, we investigated whether the specific *BRCA* mutation (*BRCA*1 vs. *BRCA*2) has a differential impact on metabolism in women.

**Methods:**

Eligible participants were *BRCA* LP/PV carriers who were premenopausal or underwent menopause –either natural or iatrogenic– within the 5 years prior to enrollment. Blood samples for lipid and glucose panels were obtained every 6 months, for a total of four time points. Body composition variables were evaluated at baseline and at the final follow-up using bioimpedance analysis. Glucose tolerance was assessed using the homeostatic model assessment for insulin resistance (HOMA-IR). Associations between lipid and glucose profile and patient characteristics were evaluated using univariable and multivariable linear regression models.

**Results:**

Fifty-seven BRCA1 and 58 BRCA2 LP/PV carriers were included in the final analysis. At baseline, *BRCA1* LP/PV carriers had a higher body mass index (BMI) (27.3 vs. 24.6 kg/m^2^, *p* = 0.01) and higher fat mass (27.3 vs. 21.9 kg, *p* = 0.013) than *BRCA2* LP/PV carriers. Insulin levels and HOMA-IR were consistently higher in *BRCA1* than in *BRCA2* LP/PV carriers at all time points, and this difference was not attributable to age, BMI, menopausal status, risk-reducing salpingo-oophorectomy, previous chemotherapy or use of cholesterol-lowering agents. The lipid profile was similar between the groups, although *BRCA1* LP/PV carriers showed a tendency toward a less favorable profile (higher LDL and lower HDL).

**Conclusions:**

These prospective results suggest that *BRCA1* LP/PV carriers might have impaired glucose tolerance and a greater tendency toward insulin resistance compared with *BRCA2* LP/PV carriers: this first report needs further independent confirmations from other cohorts.

**Supplementary Information:**

The online version contains supplementary material available at 10.1007/s10689-026-00570-3.

## Introduction

The lifetime risk of ovarian cancer (OC) by the age of 70 years is significantly elevated in women who carry likely pathogenic/pathogenic variants (LP/PV) in the *BRCA1* or *BRCA2* (*BRCA*) genes – approximately 44% for *BRCA1* and 17% for *BRCA2* mutation carriers [1]. Currently, there is no effective screening strategy to reduce OC mortality in *BRCA* LP/PV carriers. Risk-reducing salpingo-oophorectomy (RRSO) remains the only proven intervention for lowering mortality, recommended for OC prevention and detection of occult neoplasia after childbearing is complete – by the age of 35–40 years for *BRCA1* LP/PV carriers and by the age of 40–45 years for *BRCA2* LP/PV carriers [2, 3, 4, 5, 6]. Consequently, many of these women are subjected to premature surgical menopause. Early-onset breast cancer (BC), frequently diagnosed in *BRCA* LP/PV carriers, can also contribute to premature ovarian failure, particularly when chemotherapy is administered with (neo)adjuvant intent [7]. On the other hand, adjuvant endocrine treatments may include ovarian function suppression in premenopausal patients. Additionally, women with *BRCA* LP/PV have a higher risk of earlier spontaneous menopause than does the general population, especially *BRCA1* LP/PV carriers – likely because of more rapid depletion of the ovarian reserve [8, 9, 10, 11, 12, 13, 14].

Estrogens exert a beneficial effect on glucose metabolism by decreasing abdominal fat deposition, increasing lipid oxidation and energy expenditure, and improving insulin sensitivity and possibly insulin secretion [15]. Metabolic syndrome is a cluster of cardiometabolic risk factors that originate from insulin resistance, including central obesity, hypertension, low high-density lipoprotein (HDL) cholesterol, elevated serum triglycerides, and impaired glucose tolerance or overt diabetes. The menopausal transition has been associated with an increased prevalence of metabolic syndrome, particularly when menopause occurs prematurely [16, 17]. Accordingly, growing evidence suggests that women who experience premature menopause are at increased risk of developing diabetes later in life [18, 19, 20, 21]. Moreover, preclinical evidence suggests that *BRCA1* functions as an endocrine and metabolic regulator by interfering with the insulin/insulin-like growth factor 1 (IGF1) axis [22].

For this reason, we prospectively investigated whether *BRCA1* versus *BRCA2* LP/PV can differentially affect lipid and glucose metabolism in premenopausal women and those within five years of menopause.

## Materials and methods

### Study design

This single-center prospective cohort study was conducted at the Modena Family Cancer Clinic (MFCC), Italy, between November 2020 and March 2024. The MFCC is a hub center for the identification of families at increased familial or hereditary cancer risk. To date, more than 1000 women carrying a germline LP/PV predisposing to BC and/or OC have attended the MFCC for their screening programs. Regarding OC screening, women with a germline *BRCA* LP/PV undergo transvaginal ultrasound and serum CA125 testing every 6 months, and RRSO is recommended in accordance with European guidelines [3]. Eligible participants were *BRCA* LP/PV carriers who were either premenopausal or had experienced menopause – whether natural or iatrogenic (due to BC treatment or surgery) – within 5 years prior to enrollment. Women who had undergone menopause more than 5 years before the enrollment date were excluded from the study. Menopause was defined by clinical and women history criteria (natural: one year of amenorrhea, BC treatment-induced and post-RRSO). Women with personal diabetes history, also during pregnancy (gestational), have been excluded. This study was part of a larger project titled “Quality of (reproductive) life in young women at increased risk for hereditary breast and/or ovarian cancer.” The project was funded by the Italian Ministry of Health under the BANDO RICERCA FINALIZZATA 2018, with the protocol number: GR-2018‐12,367,239. The trial was approved by the Ethical Committee Area Vasta Emilia Nord (approval number: 515/2019/SPER/AOUMO, 9 July 2019). Informed consent was obtained from all recruited patients.

### Procedure

Clinical data regarding menopausal status and previous BC diagnosis and treatment were already available from medical records stored at the MFCC. At baseline and during follow-up, women were asked about all medications and supplements they regularly used, with particular attention to aromatase inhibitors, LH-RH analogs, tamoxifen, cholesterol- and glucose-lowering agents. Hormone replacement therapy (HRT) use at baseline was recorded due to its potential metabolic effects. In particular, tibolone was prescribed to women who had undergone premature menopause following RRSO and had no prior history of breast cancer.

A blood sample to assess lipid and glucose profiles (including total cholesterol, HDL cholesterol, LDL cholesterol, triglycerides, insulin, and glucose) was obtained every 6 months, for a total of four time points (baseline, 6th month, 12th month, and 18th month). All blood samples were collected in the morning after an 8-hour fast, and all analyses were conducted in the same laboratory. Glucose was measured using the hexokinase enzymatic method (Beckman Coulter, Brea, CA, USA). Plasma cholesterol (total, HDL, and LDL) and triglycerides were assessed using enzymatic colorimetric methods (Beckman Coulter). Insulin levels were determined by chemiluminescence immunoassay using the LIAISON^®^ XL Analyzer. Glucose tolerance was evaluated using the homeostatic model assessment for insulin resistance (HOMA-IR), calculated as [fasting glucose (mg/dL) × fasting insulin (mU/L)] / 405 [23].

Body mass index (BMI) and body composition variables (fat mass, fat-free mass, and total body water) were assessed at baseline and at the final follow-up using bioimpedance analysis (BIA). A digital column bioimpedance analyzer was used to measure segmental body composition (whole body, right arm, left arm, right leg, left leg) with a multi-frequency, eight-electrode arrangement (measurement frequencies: 5 kHz / 50 kHz / 250 kHz; measurement range: 75–1500 Ohm; TANITA Corporation, Tokyo, Japan). During the assessment, the patient stood upright with bare feet placed on four stainless steel footpad electrodes and held a handlebar equipped with two stainless steel electrodes on each hand. The reliability and validity of this method for estimating human body composition have been supported by previous studies [24].

### Statistics

The primary outcome of this study was to evaluate differences in the lipid and glucose metabolic profiles of women carrying *BRCA1* versus *BRCA2* LP/PV over an 18-month period. Numerical variables are reported as means and standard deviations, while categorical variables are expressed as absolute frequencies and percentages. The association between outcome measures and patient characteristics was assessed using univariable and multivariable linear regression models. To examine changes in outcome measures over time (i.e. at each time point) and their association with *BRCA* LP/PV status, multivariable linear mixed-effects models were estimated. These models included age, BMI, prior chemotherapy for BC, menopausal status, type of menopause, and use of cholesterol-modifying agents as covariates, in order to adjust the estimation of the relationship between *BRCA* LP/PV and the metabolic profile. A random intercept term was included in the mixed-effects models to account for repeated measures on the same individuals. Results are expressed as mean differences with corresponding 95% confidence intervals and *p*-values. Statistical significance was defined as a p-value of < 0.05. All analyses were conducted using R software, version 4.3.2 (The R Foundation for Statistical Computing, 2023).

This was a pilot study then it was not conducted for the purpose of inference, then a specific power calculation has not been conducted.

## Result

A total of consecutive 119 BRCA LP/PV carriers were included in the study: 2 (1.7%) were lost to follow up for consent withdrawal and 2 (1.7%) were diagnosed with breast and ovarian cancer respectively during follow-up period and then excluded from the final analysis.

### Baseline characteristics

For these reasons, 115 women were included in the final analysis: 57 *BRCA1* and 58 *BRCA2* LP/PV carriers. Their baseline demographic characteristics are shown in Table 1. *BRCA1* LP/PV carriers were significantly younger than *BRCA2* LP/PV carriers (48.3 vs. 50.9 years, *p* = 0.048). The two groups were balanced in terms of menopausal status (68.4% *BRCA1* vs. 69.0% BRCA2); however, *BRCA1* LP/PV carriers experienced menopause at a significantly younger age (45.2 vs. 47.9 years, *p* = 0.020) and more frequently underwent iatrogenic menopause (74.4% vs. 52.5%, *p* = 0.044) compared with *BRCA2* LP/PV carriers. A history of chemotherapeutic treatment for BC was more common among *BRCA1* than *BRCA2* LP/PV carriers (42.1% vs. 15.5%, *p* = 0.002). Vice versa, an ongoing adjuvant hormonal treatment with aromatase inhibitors, LH-RH analogs and/or tamoxifen for BC was more frequent among BRCA2 than BRCA1 LP/PV carriers (13.8% vs. 1.7%, *p* = 0.016). At baseline, 9 women were receiving HRT with tibolone (5 *BRCA1* and 4 *BRCA2* LP/PV carriers), with no significant difference between the groups. The proportion of patients using cholesterol-lowering agents did not differ between the groups, and no participants were diabetic or taking hypoglycemic medications.

**Table 1:** Demographic features of included women at baseline (crude analysis)

	*BRCA1* (*n* = 57)	*BRCA2* (*n* = 58)	*p*-value
	Mean (SD), median	Mean (SD), median	
Age at enrolment (years)	48.3 (7.2), 46.3	51.0 (7.2), 49.6	**0.048**
Age at menopause (years)	45.2 (5.6), 45.0	47.9 (4.4), 47.0	**0.020**
	*n*,* %*	*n*,* %*	
Post menopause	39/57 (68.4%)	40/58 (69.0%)	0.950
Type of menopause *Natural* *Iatrogenic* *BC treatment-induced* *Post-RRSO*	10/39 (25.6%) 29/39 (74.4%) 7/39 (18.0%) 22/39 (56.4%)	19/40 (47.5%) 21/40 (52.5%) 4/40 (10.0%) 17/40 (42.5%)	**0.046** *
Prior BC	32/57 (56.1%)	25/58 (43.1%)	0.163
Prior chemotherapy	24/57 (42.1%)	9/58 (15.5%)	**0.004**
RRSO	34/57 (59.6%)	36/58 (62.1%)	0.790
HRT (Tibolone)	5/57 (8.8%)	4/58 (6.9%)	0.708
Use of cholesterol-lowering agents	17/57 (29.8%)	12/58 (21.0%)	0.259
Use of aromatase inhibitors, LH-RH analogs, tamoxifen	1/57 (1.7%)	8/58 (13.8%)	**0.016**

* natural vs. iatrogenic. Abbreviations: BC: breast cancer; HRT: Hormone Replacement Therapy; LH-RH: Luteinizing Hormone-Releasing Hormone; MD: mean difference; RRSO: risk-reducing salpingo-oophorectomy; SD: standard deviation. Bold means statistically significant

Despite their younger age, at enrollment *BRCA1* LP/PV carriers had a higher BMI (27.3 vs. 24.6 kg/m^2^, *p* = 0.01) and greater fat mass (27.3 vs. 21.9 kg, *p* = 0.013) than *BRCA2* LP/PV carriers, while fat-free mass and total body water did not statistically differ among the two groups (Supplemental Fig. 1, Table 2). Differences in BMI and fat mass between *BRCA1* and *BRCA2* LP/PV carriers remained stable over the study period as confirmed at the final follow-up measurement at 18 months (Table 2, Supplemental Fig. 1).

**Table 2:** Body composition variables at the baseline and at the last follow-up

	Baseline			Last follow-up (18 months later)		
	BRCA1 (*n* = 57)	BRCA2 (*n* = 58)		BRCA1 (*n* = 57)	BRCA2 (*n* = 58)	
	*Mean (SD)*,* median*		*p-value*	*Mean (SD)*,* median*		*p-value*
BMI (kg/m^2^)	27.3 (6.2), 26.0	24.6 (4.8), 23.3	**0.011**	27.6 (5.9), 26.0	24.8 (4.6), 24.1	**0.006**
Fat mass (kg)	27.3 (13.1), 23.8	21.9 (9.2), 19.3	**0.013**	26.8 (12.0), 24.2	20.9 (8.3), 19.2	**0.005**
Fat-free mass (kg)	46.7 (6.1), 45.7	44.9 (4.8), 44.2	0.103	47.4 (5.7), 46.7	45.7 (4.7), 44.1	0.095
Total body water (kg)	34.2 (4.4), 33.5	33.1 (3.2), 32.5	0.145	34.7 (4.2), 34.2	33.4 (3.4), 32.2	0.094

BMI: body mass index; MD: mean difference; SD: standard deviation. Bold means statistically significant

### Glucose metabolism

**Fig. 1:**
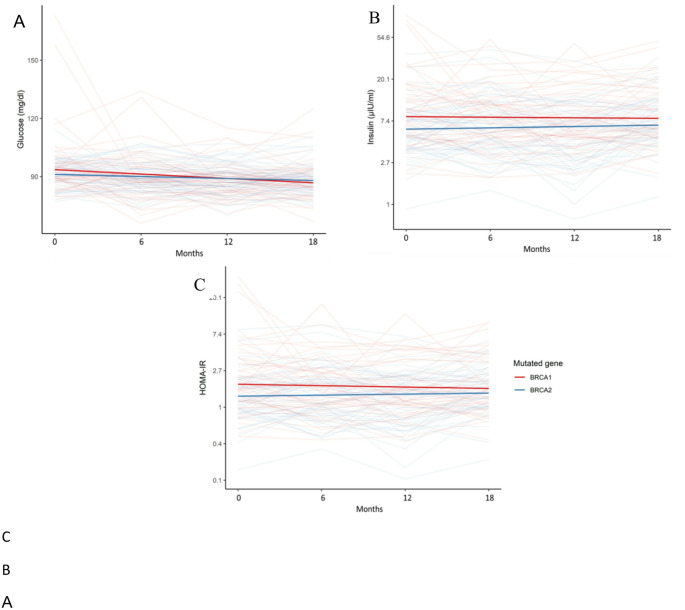
Evolution over time of glucose metabolism (crude values): **A** fasting glucose, **B** insulin and **C** HOMA IR (*BRCA1* *n* = 57, *BRCA2* *n* = 58)

In the crude analysis at baseline, women with *BRCA1* LP/PV had higher insulin levels (13.4 vs. 7.8 µIU/mL, *p* = 0.03) than *BRCA2* LP/PV carriers, while no differences were observed in glucose or HOMA-IR (Table 3). After adjusting for age, BMI, menopausal status, prior chemotherapy, and use of cholesterol-modifying agents, insulin levels and HOMA-IR were independently higher in *BRCA1* than *BRCA2* LP/PV carriers (Table 4). These differences in glucose metabolism persisted over time during the entire follow-up (Fig. 1).

**Table 3:** Basal glucose and lipid metabolism profile (crude analysis)

	*BRCA1* (*n* = 57)	*BRCA2* (*n* = 58)	BRCA2 versus BRCA1	*p*-value
	Mean (SD), median	Mean (SD), median	MD (95%CI)	
Insulin (µIU/ml)	13.4 (18.2), 8.0	7.8 (6.5), 5.9	-5.6 (-10.6:-0.6)	**0.030**
Glucose (mg/dl)	94.9 (15.9), 91	91.4 (7.7), 93.0	-3.5 (-8.0:1.1)	0.139
HOMA-IR	3.7 (6.6), 1.6	1.8 (1.6), 1.4	-1.9 (-3.6:-0.1)	**0.039**
Total cholesterol (mg/dl)	212.7 (36.2), 211.0	211.91(41,3), 206.0	-0.8 (-15.0:13.4)	0.916
HDL cholesterol (mg/dl)	63.8 (14.7), 63.0	68.8 (25.5), 65.0	5.0 (-2.7:12.6)	0.204
LDL cholesterol (mg/dl)	136.3 (29.3),130.0	130.7 (32.3), 123.0	-5.7 (-17.0:5.6)	0.328
Triglycerides (mg/dl)	92.1 (46.7), 82.0	86.3 (36.3), 83.0	-5.8 (-21.1:9.45)	0.457

HDL: high density lipoproteins; HOMA-IR: the homeostatic model assessment for insulin resistance; LDL: low density lipoproteins; MD: mean difference; SD: standard deviation. Bold means statistically significant

**Table 4:** Glucose and lipid metabolism profile, at baseline and change over time (covariate analysis)

		*BRCA2* versus *BRCA1* MD (95%CI)	*p*-value
Insulin (µIU/ml)	Baseline	-6.95 (-13.01:-0.89)	**0.027**
	Change over time	0.46 (-1.44:2.36)	0.637
Glucose (mg/dl)	Baseline	-5.63 (-12.00:0.73)	0.085
	Change over time	1.06 (-1.15:3.27)	0.349
HOMA-IR	Baseline	-2.70 (-4.66:-0.73)	**0.008**
	Change over time	0.39 (-0.32:1.10)	0.286
Total cholesterol (mg/dl)	Baseline	3.94 (-17.21:25.09)	0.716
	Change over time	-1.97 (-7.19:3.25)	0.460
HDL cholesterol (mg/dl)	Baseline	9.69 (-0.98:20.36)	0.079
	Change over time	-1.01 (-3.76:1.75)	0.476
LDL cholesterol (mg/dl)	Baseline	-4.71 (-21.30:11.88)	0.579
	Change over time	-0.55 (-4.95:3.84)	0.805
Triglycerides (mg/dl)	Baseline	-5.30 (-26.91:16.31)	0.632
	Change over time	-6.56 (-12.82:-0.31)	0.041

Bold means statistically significant

### Lipid metabolism

**Fig. 2:**
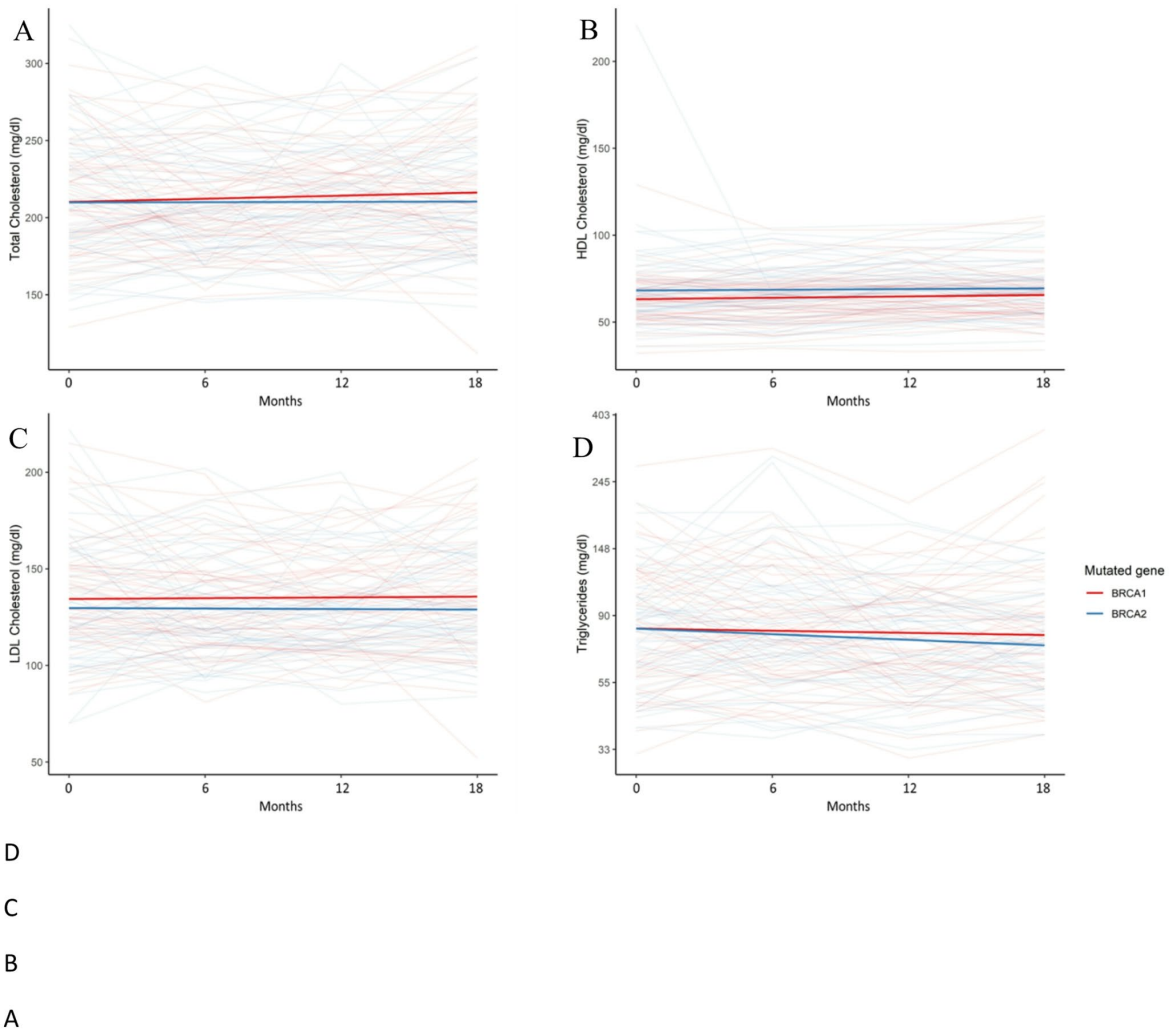
Evolution over time of lipid metabolism (crude values): **A** total cholesterol, **B** HDL cholesterol, **C** LDL cholesterol and **D** triglycerides (*BRCA1* *n* = 57, *BRCA2* *n* = 58)

In the crude analysis at baseline, total cholesterol and triglycerides were similar between *BRCA1* and *BRCA2* LP/PV carriers. Although not statistically significant, HDL and LDL cholesterol tended to be lower and higher, respectively, in *BRCA1* vs. *BRCA2* LP/PV carriers (Table 3). After adjusting for age, BMI, menopausal status, prior chemotherapy, and use of cholesterol-lowering or cholesterol-increasing (aromatase inhibitors, LH-RH analogs and/or tamoxifen) agents, the lipid profile was confirmed to not statistically differ between *BRCA1* and *BRCA2* LP/PV carriers in the covariate analysis, although *BRCA1* LP/PV carriers tended to exhibit a less favorable profile, with higher LDL and lower HDL cholesterol levels (Fig. 2; Table 4).

## Discussion

In this prospective cohort study, we found that *BRCA1* LP/PV carriers had higher insulin levels and HOMA-IR than *BRCA2* LP/PV carriers, independent of menopausal status, age, BMI, or BC therapies. This finding was consistent throughout 18 months of follow-up. These results suggest that *BRCA1* LP/PV carriers might have impaired glucose tolerance and a greater tendency toward insulin resistance compared with *BRCA2* LP/PV carriers. Despite the higher BMI observed in *BRCA1* LP/PV carriers, we did not find significant differences in lipid profiles between the groups, although HDL levels tended to be lower and LDL levels higher in *BRCA1* LP/PV carriers.

Although *BRCA1* and *BRCA2* LP/PV carriers are often put together under the umbrella of the Hereditary Breast and Ovarian Cancer (HBOC) syndrome, they have distinct features. *BRCA1* LP/PV carriers have a higher risk of OC at an earlier age than *BRCA2* ones, so RRSO is recommended approximately five-eight years before in *BRCA1* LP/PV carriers [1, 2, 3, 4, 5, 6]. Considering the risk of BC, *BRCA1* LP/PV carriers are prone to develop early-onset and triple-negative BC that frequently requires chemotherapy administration with (neo)adjuvant intent.^7^ Both these factors can contribute to premature iatrogenic menopause in *BRCA1* LP/PV carriers. By the other hand, hormonal-receptor positive BC is most common among *BRCA2* LP/PV carriers [25]. The baseline characteristics of our cohort reflect the distinct features of *BRCA1* and *BRCA2* LP/PV carriers. Indeed, *BRCA1* LP/PV carriers experienced menopause at a significantly younger age and more frequently underwent iatrogenic menopause compared with *BRCA2* LP/PV carriers. A history of chemotherapeutic treatment for BC was most common among *BRCA1* LP/PV carriers, while an ongoing adjuvant hormonal treatment for BC was most frequent among *BRCA2* LP/PV carriers.

The association between glucose metabolism and *BRCA* LP/PV has been previously examined in two retrospective cohort studies. Bordeleau et al. [26] reported a two-fold increased risk of diabetes among *BRCA* LP/PV carriers in the 15 years following a BC diagnosis, particularly in women with a BMI of > 25.0 kg/m^2^ at the time of diagnosis and who were treated with chemotherapy. However, no difference in diabetes incidence was observed before BC diagnosis compared with healthy controls. Notably, nearly 75% of the patients enrolled were *BRCA1* LP/PV carriers. Oliveiro et al. [27] enrolled 438 women (269 *BRCA1* LP/PV carriers and 169 *BRCA2* LP/PV carriers), more than 80% of whom had a previous diagnosis of BC and/or OC. They found that *BRCA loss-of-function* LP/PV (e.g. frameshift, nonsense, large deletion and spliceogenic variants) were associated with higher plasma glucose and serum insulin levels than nonsynonymous (e.g. missense and small in-frame deletions) LP/PV. In both studies, a direct comparison between *BRCA1* and *BRCA2* LP/PV carriers was not performed.

Being carriers of *BRCA* LP/PV involves several health concerns, primarily related to BC and OC prevention. However, preclinical studies analyzing the role of *BRCA1* as a tumor-suppressor gene have revealed a close interaction between *BRCA1* and the insulin/IGF1 axis. Specifically, IGF1 contributes to BC initiation and progression, and the tyrosine kinase domain of the IGF1 receptor (IGF1R) is constitutively activated in many cancer cells [28, 29, 30, 31]. The BRCA1 protein counteracts this by repressing *IGF1R* transcription and promoter activity, as demonstrated in breast, prostate, and endometrial cancer cells [32, 33, 34, 35, 36, 37, 38, 39]. As a result, primary BCs derived from *BRCA1* LP/PV carriers express higher levels of IGF1R than sporadic cases [40]. However, the implications of *BRCA1* LP/PV on lipid and glucose metabolism remain poorly investigated. Preclinical models in BC cells have shown that wild-type BRCA1 inhibits glycolysis, activates the tricarboxylic acid cycle and oxidative phosphorylation, and reduces levels of ketone bodies and free fatty acids [41]. Moreover, *BRCA1* mutant cells exhibit increased lipogenesis [42].

The strength of our study lies in its prospective design and the availability of repeated measures over an 18-month follow-up, which confirmed the baseline observations. Additionally, women were questioned about all medications and supplements taken, helping to minimize potential biases related to the concomitant use of glucose- and/or cholesterol-modifying agents. A matched analysis between *BRCA1* and *BRCA2* LP/PV carriers was explored; however, only a limited subset of patients could be matched for key clinical variables, resulting in a substantial reduction in sample size and statistical power. Furthermore, such an approach would not have allowed adjustment for all relevant covariates included in our models, such as lipid-modifying therapies. Therefore, we considered multivariable regression models to be a more appropriate approach for controlling confounding in this study.

However, our study has several limitations. First, although at least 8 h of fasting were recommended prior to blood sampling, we could not ensure that all participants adhered to this instruction. This might have influenced glucose and insulin levels reported in both *BRCA1* and *BRCA2* LP/PV carriers. Secondly, the observed reduction in glucose levels among *BRCA1* LP/PV carriers during follow-up remains only partially explained. It is plausible that increased dietary awareness—such as reduced intake of simple sugars—or enhanced physical activity may have contributed to this finding. These factors, combined with the small sample size and single-center design, limit the generalizability of our results regarding glucose tolerance and insulin resistance, underscoring the need for larger, multicenter cohort studies. As regards body composition analysis, BIA relies on prediction equations derived from large populations to estimate body composition parameters. While this method is effective for rapid, quantitative assessments in fitness settings, its accuracy may be limited in clinical or research contexts. In such cases, Bioelectrical Impedance Vector Analysis (BIVA provides a more nuanced evaluation of hydration status, cellular health, and body composition [43]. Consequently, standard BIA may have overlooked some aspects of body composition in our study. Finally, our study lacks a control arm of BRCA-wildtype women, which would have helped confirm and strengthen our findings: we are designing a new prospective study including a control group of BRCA wild-type women and a male BRCA group.

Our work has several potential clinical implications. Insulin resistance and hyperinsulinemia typically precede the onset of diabetes by 10 to 20 years [44, 45]. If the *BRCA1* LP/PV itself is a risk factor for impaired glucose tolerance, endocrinologic counseling may be particularly beneficial for *BRCA1* LP/PV carriers to help reduce the risk of diabetes and metabolic syndrome – especially in women undergoing premature ovarian failure or iatrogenic menopause due to RRSO. Beyond lowering cardiovascular risk, it is also plausible to speculate that reducing insulin levels could help decrease the risk of BC in women carrying a *BRCA* LP/PV.

## Conclusion

Our study found that women carrying *BRCA1* LP/PV might have impaired glucose tolerance compared with *BRCA2* LP/PV carriers, regardless of age, BMI, menopausal status, RRSO status, or previous chemotherapy for BC. Larger multicenter cohort trials are ultimately needed to confirm these findings and to identify strategies for reducing the risk of metabolic syndrome, particularly in women with *BRCA1* LP/PV.

## Electronic supplementary material

Below is the link to the electronic supplementary material.

Supplementary file 1 (DOCX 460 kb)

## Data Availability

No datasets were generated or analysed during the current study.
