# Supplementary material for: Impaired glucose tolerance in women with BRCA1 versus BRCA2 pathogenic or likely pathogenic variants: Results from a prospective cohort study
Source: Fam Cancer. 2026 May 14;25(2):55. doi: 10.1007/s10689-026-00570-3 (PMC13176168; doi:10.1007/s10689-026-00570-3)

**Supplemental Figure**

**Supplemental Figure 1** Basal body composition. Mean values of body mass index (BMI), fat mass, fat-free mass and total body water at the baseline (BRCA 1 n=57 vs. BRCA 2 n=58 women).


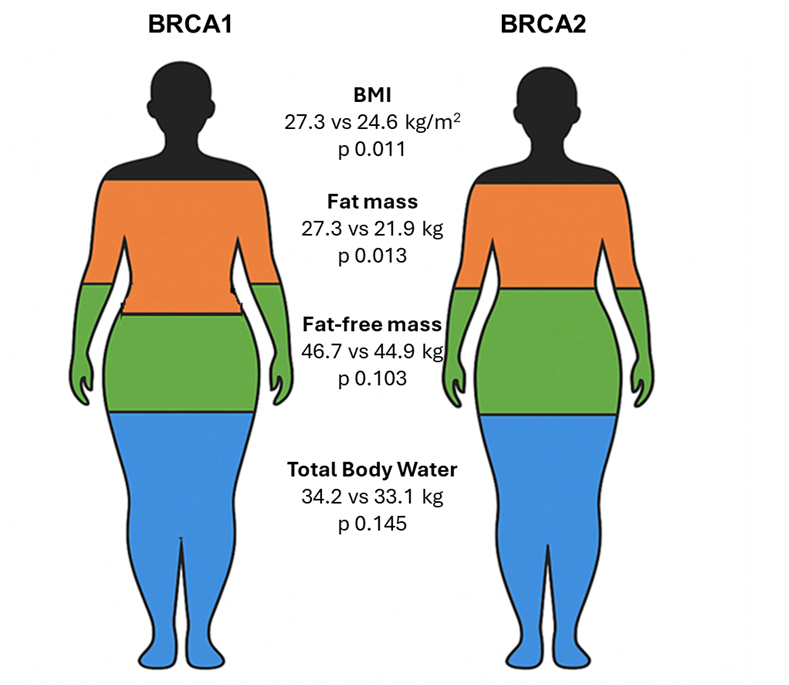

Supplement: Supplementary file 1 — (DOCX 460 kb) [file 10689_2026_570_MOESM1_ESM.docx]
